# Nanogap Electrode-Enabled Versatile Electrokinetic Manipulation of Nanometric Species in Fluids

**DOI:** 10.3390/bios12070451

**Published:** 2022-06-24

**Authors:** Qiang Zhao, Yunjiao Wang, Bangyong Sun, Deqiang Wang, Gang Li

**Affiliations:** 1 Key Laboratory of Optoelectronic Technology and Systems, Ministry of Education, Defense Key Disciplines Lab of Novel Micro-Nano Devices and System Technology, Chongqing University, Chongqing 400044, China; qiangzh@cqu.edu.cn (Q.Z.); 20152602015@cqu.edu.cn (B.S.); 2 Chongqing Key Laboratory of Multi-Scale Manufacturing Technology, Chongqing Institute of Green and Intelligent Technology, Chongqing 400714, China; wangyunjiao@cigit.ac.cn

**Keywords:** nanogap electrode, microfluidics, dielectrophoresis, nanomanipulation

## Abstract

Noninvasive manipulation of nanoscopic species in liquids has attracted considerable attention due to its potential applications in diverse fields. Many sophisticated methodologies have been developed to control and study nanoscopic entities, but the low-power, cost-effective, and versatile manipulation of nanometer-sized objects in liquids remains challenging. Here, we present a dielectrophoretic (DEP) manipulation technique based on nanogap electrodes, with which the on-demand capturing, enriching, and sorting of nano-objects in microfluidic systems can be achieved. The dielectrophoretic control unit consists of a pair of swelling-induced nanogap electrodes crossing a microchannel, generating a steep electric field gradient and thus strong DEP force for the effective manipulation of nano-objects microfluidics. The trapping, enriching, and sorting of nanoparticles and DNAs were performed with this device to demonstrate its potential applications in micro/nanofluidics, which opens an alternative avenue for the non-invasive manipulation and characterization of nanoparticles such as DNA, proteins, and viruses.

## 1. Introduction

The manipulation of nanoscopic species in liquids has attracted considerable attention due to its potential applications in diverse fields [1,2,3,4,5]. In particular, the ability to concentrate, sort, and separate nanoparticles is highly desirable for a variety of practical functionalities [6]. Despite the increasing demand, the manipulation of nanoparticles is extremely challenging due to the predominant effect of Brownian motion at the nanoscale. A variety of approaches have been developed to manipulate nanoparticles using external fields including optical, magnetic, fluidic, centrifugal, and electrical biases [7,8,9,10,11,12,13,14,15,16,17]. Among these approaches, electric actuation has gained special attention due to the ease of implementation and its suitability in miniaturization [18,19,20]. Recently, electrokinetics has been widely applied to various applications in the manipulation of biological and synthetic microparticles in microfluids, which include trapping, sorting, and transporting [21,22,23]. To manipulate smaller objects (e.g., nanoparticles, the electric field must be increased to offset the decline in electrokinetic forces caused by the size decrease in the particles, often resulting in an undesirable Joule heating effect [24]. To address this problem, an alternative strategy is to reduce the interelectrode gap or the local cross section of the channel to the nanoscale, inducing strongly enhanced fields around the nanogap or the channel nano-constriction [25]. Nanogap electrodes and nanoconstriction structures have been successfully demonstrated in the trapping nanoparticles and biomolecules [26,27]. However, the use of nanostructured devices as a versatile tool to enrich and sort nanoparticles has not been demonstrated. Additionally, the requirement for complicated and expensive nanofabrication instruments (e.g., focused ion beam and atomic layer deposition) also makes the nanostructured devices inaccessible to a large number of researchers.

In this paper, we report on a novel electrokinetically controlled nano-manipulation technique based on a cost-effective micro/nanohybrid device. In this device, an integrated nanogap-electrode architecture facilitates the generation of strong dielectrophoretic forces at low voltages, effectively capturing and spatiotemporally manipulating nanoparticles and biomacromolecules. Unlike conventional nanoscale devices produced by complicated and expensive nanolithography, our device, created by transversely embedding swelling-induced nanogap electrodes in a microfluidic channel, can be conveniently fabricated using standard photolithographic processes, facilitating a more economical approach to achieve nano-manipulations. The capabilities of the device were validated by performing the trapping, enriching, and sorting of the nanoparticles and DNAs. Due to its versatility and cost-effectiveness, this approach will be useful across a wide range of applications in nanoparticle assembly, biomarker detection, and single-molecule studies.

## 2. Materials and Methods

### 2.1. Device Fabrication

The entire process to fabricate nanogap electrodes in a microchannel mainly includes three steps: fabricating the nanogap electrodes, fabricating the PDMS microchannels, and then bonding the two components. To fabricate the nanogap electrodes in this study, a layer of epoxy (crystal epoxy 155, Ausbond Co., Ltd., Shenzhen, China) was spin-coated on a glass substrate at 2000 rpm for 40 s. After spin-coating, the sample was given a relaxation time of 30 min at 25 °C to allow for reflowing of the coated epoxy and then cured at 110 °C for 6 h. Next, photolithography with the positive photoresist (EPG 535, Everlight Chemical Industrial Corporation, Taiwan, China) was performed over the epoxy to define the pattern of the metal electrodes. Cr and Au were alternately sputtered on the patterned epoxy substrate with a structure of 25/25/25/25 nm by magnetron sputtering (DP650, Alliance Concept, Cran Gevrier, France). After metal sputtering, a lift-off process was conducted to obtain the patterned electrodes. Subsequently, the sample was immersed in 60% aqueous acetone for 20 min followed by pure acetone for 20 s to induce cracks in the patterned metal layer and thus obtain metal nanogaps. After the attainment of the metal cracks, the sample was placed in a vacuum environment of −90 kPa for 30 min to remove the organic molecules permeated in the epoxy. PDMS microchannels were molded using soft lithography. A layer of EPG 535 was spin-coated on a glass substrate at 500 rpm for 10 s followed by 1000 rpm for 30 s. For the fabrication of a 4.1-μm-deep channel, the spinning speed was set to 500 rpm for 40 s. After baking at 95 °C for 5 min, standard photolithography was performed to obtain micro molds. The molds were then cast with PDMS (Sylgard 184, 10:1) from Dow Corning (Midland, MI, USA) and cured at 70 °C for 4 h to obtain the microchannels. The PDMS microchannels were then punched to obtain inlets and outlets of 2 mm in diameter. Finally, the molded PDMS and metal-patterned epoxy substrate were aligned and bonded by the following process [28,29]. The epoxy substrate containing nanogap electrodes was treated by oxygen plasma and exposed to 3-mercaptopropyl-trimethoxysilane under a vacuum pressure of −95 kPa for 1 h. Subsequently, the PDMS slab containing microchannels was aligned and bonded to the metal-patterned epoxy substrate coated with a self-assembled monolayer of MPT using an alignment system of lithography (ABM/6/350/NUV/DCCD/BSV/M, ABM, Inc., Silicon Valley, CA, USA). After the bonding process, a standing time of over 8 h was required for high bonding reliability.

### 2.2. PS Particles and λ-DNA Preparation

Two different sizes of carboxylate-modified fluorescent polystyrene (PS) particles (PSGF00200 and PSRF00020, 10 mg/mL, Beijing Zhongkeleiming Technology Co., Ltd., Beijing, China), 200 nm and 20 nm in diameter, respectively, were treated by centrifugation (TG16-W, Changsha Xiangzhi Centrifuge Instrument Co., Ltd., Changsha, China) at 6000 rpm for 10 min and then dispersed in DI water. To investigate the influence of the channel height, peak-to-peak voltage (V*_pp_*), and frequency on DEP manipulation, the PS particles were diluted to 10 μg/mL (1:1000). For the negative DEP (N-DEP) and high-speed enrichment processes, the dilution ratio of the particles 200 nm in diameter was 1:100 and 1:200, respectively. For the DEP sorting process, nanoparticles were blended at a ratio of 1:1 and then diluted at a ratio of 1:10. For the filtration of the particles, the dilution ratio of the 200 nm and 20 nm particles was 1:1000 and 1:10, respectively, to obtain similar fluorescence intensity. Next, the diluted nanoparticles were blended at a ratio of 1:1. The conductivity was measured by a conductivity analyzer (*σ_f_* = 0.001 S/m, DDS-11A, Inesa Scientific Instrument Co., Ltd., Shanghai, China). For the λ-DNA trapping process, the λ-DNA stock solution (48.5 kbp, 500 μg/mL, 0.1 mL, New England Biolabs, Inc., Ipswich, MA, USA) was diluted by 1 × TE buffer (0.03 S/m) at a ratio of 1:50. Then, the prepared solution was mixed with a YOYO-1 dye solution (1 mM, 15 μL, Thermo Fisher Scientific, Eugene, OR, USA) and incubated for 60 min at room temperature. After incubation, the solution was further diluted at a ratio of 1:100 (0.1 μg/mL). For the DNA sorting experiment, the PBR322 solution (4.26 kbp, 200 μg/mL, 20 μL, Beijing Solarbio Science & Technology Co., Ltd., Beijing, China) was diluted by 1 × TE buffer in a ratio of 1:20, followed by mixing with a POPO-3 dye solution (1 mM, 3 μL, Thermo Fisher Scientific, Eugene, OR, USA) and incubation. Finally, the POPO-3 stained PBR322 (10 μg/mL) solution was mixed with YOYO-1 stained λ-DNA (10 mg/mL) at a ratio of 2:1.

### 2.3. Vacuum-Assisted Injection and Driving of the Dispersion

In the manipulation process of the PS particles, the microchannel chips were degassed in a vacuum environment of −90 kPa for 30 min and then filled with deionized (DI) water (2.5 μL in the inlet and outlet). After the completion of the microchannel filling, the DI water in the inlet was replaced by particle suspensions (2.5 μL). For the high-speed DEP portion of the parameter study, another drop of suspension (2.5 μL) was added to the inlet. The flow rate in the microchannel was measured as 29 μm/s. For the low-speed N-DEP enriching and sorting experiments, the added suspension volume was 0.2 μL (the flow rate was measured as 1 μm/s). In the filtration process, a drop of suspension (2.5 μL) was added to the inlet and the DI water was removed from the outlet of the microchannel. The switch of flow direction was realized by the displacement of DI water from one outlet to another.

For the manipulation of DNAs, the degassed microchannel chips were filled with TE buffer (2.5 μL in the inset and outlet). The buffer in the inlet was then replaced by the DNA suspension (3.7 μL). The flow rate of DNA was measured as 7 μm/s.

### 2.4. DEP Manipulation and Observation

The setup of the system for performing and recording nanogap electrode-enabled dielectrophoretic manipulation of the nanoparticles is shown in Figure S1. An AC voltage was applied to the nanogap electrodes by an arbitrary function generator (AFG1062, Tektronix, Beaverton, OR, USA). The real-time signal was monitored by an oscilloscope (TDS2014B, Tektronix, Beaverton, OR, USA) to ensure a stable frequency and V*_pp_*. The fluorescence image was collected with 20×, 0.4 NA and 10×, 0.25 NA objectives. The excitation light wavelengths were 470 nm for green fluorescence and 535 nm for red fluorescence. During the experiment, the excitation light illuminated the nanogaps in the microchannel. The excited fluorescence of nanoparticles was collected by the CCD camera (MTR3CCD01400KPB, Suzhou Jingtong Instrument Co., Ltd., Suzhou, China). The frame rate was set to 15 frames/s. The connected images were measured using ToupView software. In the DNA trapping experiment, the image enhancement using the software was set to 3000% to observe a single molecule.

## 3. Results

### 3.1. Concept of the Micro/Nanohybrid Device 

**Figure 1:**
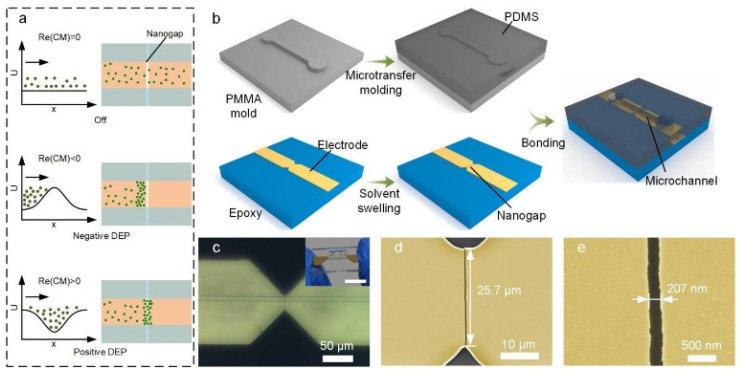
Nanogap-electrode-enabled DEP system. (**a**) Concept of nanogap-electrode-enabled versatile DEP manipulation. (**b**) Schematic illustration of the fabrication process of the nanogap electrodes/microchannel composite structure. (**c**) Microscopic image of the composite structure. Inset: photograph of the bonded device. Scale bar, 10 mm. (**d**,**e**) SEM of the nanogap electrode structure (yellow). A crack initiates from the sharp notch and ends at the other notch.

In the proposed micro/nanohybrid device, when an AC voltage is applied to the nanogaps, the forces experienced by suspended nanoparticles near the nanogap electrode include DEP force, Stokes drag force, gravity force, and buoyancy force. Assuming that the buoyancy force balances out the gravity force, the nanoparticle velocity (**u**_Total_) can be expressed as **u**_Total_ = **u**_Fluid_ + **u**_DEP_, where **u**_DEP_ is the velocity of the nanoparticles driven by DEP relative to the fluid (Note S1, Supplementary Materials) and the **u**_Fluid_ is the velocity of the fluid affected by AC electroosmosis (ACEO) and AC electrothermal (ACET) (Note S2, Supplementary Materials) [30,31,32,33]. Among these forces, the DEP force generated by the nanogaps can attract or repel the suspended nanoparticles near the nanogap, resulting in an ideal manipulation method to trap and sort the nanoparticles. Figure 1a depicts the basic principle of our device for the versatile manipulation of nanoparticles suspended in a fluid. According to classical DEP theory, the time-averaged DEP force acting on a polarizable particle in a non-uniform field is given by F_DEP_ = 2πr^3^ε_m_Re(CM)∇E^2^, where r is the particle radius, ε_m_ is the absolute permittivity of the surrounding medium, E is the root-mean-squared value of the AC alternating current (AC) electric field at the position of the particle, and Re(CM) is the real part of the Clausius–Mossotti (CM) factor, representing the effective polarizability of the particle in a medium [34,35,36]. By varying the frequency of the applied voltage, the nanogap electrodes can tailor the potential energy profile experienced by the nanoparticles in the fluid and thus noninvasively control their motion. Depending on the desired potential energy profile to be experienced by the suspended nanoparticles, the device can be operated in three modes: opening, damming, and trapping. In opening mode, no voltage is applied to the nanogap electrodes; thus, the suspended nanoparticles do not experience the dielectrophoretic force and can freely pass over the nanogap electrode. In damming or trapping mode, a tailored AC voltage is applied to the nanogap electrode to generate a sufficiently strong N-DEP or positive DEP (P-DEP) force on the suspended nanoparticles, which forms a potential barrier to block them or a potential well to trap them.

Based on this principle, we have developed a fabrication process to construct a nanogap-electrode-based DEP control unit that is transversely embedded at the bottom of a microfluidic channel to perform nanoparticle manipulation (Figure 1b). To achieve economical DEP nano-manufacturing, we adopted a swelling-induced cracking technique to fabricate the nanogap electrodes, which simply combined the photolithographic metal patterning and organic solvent swelling without the requirement of complicated and expensive nanofabrication equipment. The fabrication process of the nanogap electrodes was adopted from our previous work with some modifications [37]. First, a layer of polymer (epoxy) was spin-coated on a glass substrate. Next, Cr and Au multilayers with microscale notches were patterned on the epoxy substrate by traditional photolithography and the lift-off process. The sample was subsequently immersed in aqueous acetone to swell the epoxy and induce a crack in the patterned metal layer. After the attainment of the metal crack, the sample was placed in a vacuum environment to remove the organic molecules permeated in the epoxy, reducing the gap width to the nanoscale. Finally, the substrate containing the prepared nanogap electrodes was aligned and bonded to a PDMS slab containing a microchannel (Figure S2). The nanogap was designed to be perpendicular to the microchannel in order to avoid the movement of nanoparticles along the nanogaps. Furthermore, the nanogap perpendicular to the microchannel can also provide the maximum DEP force against the Stokes force from the fluid. In our study, the nanogap had a width of 207 nm, a depth of 100 nm, and a length of 25.7 μm (Figure 1d,e). Benefitting from the ultra-short interelectrode distance (~200 nm), the nanogap electrodes could generate an electric field up to 10^7^ V/m and an ∇E^2^ value of up to 10^21^ V^2^/m^3^ around the nanogap when applying an AC voltage of 5 V, enabling the effective manipulation of the nanoparticles (Figure S3).

### 3.2. Analysis of the Effective Working Range and Frequency Properties of the Unit 

Since ∇**E**^2^ weakens moving away from the nanogaps, the DEP force generated by the nanogap electrodes dominates only in a small area around the nanogaps (Figure S4). To achieve effective and reliable DEP nano-manipulation, the depth of the microchannel and the voltage applied to the nanogaps must be optimally designed to confine the trajectory of the nanoparticles within the effective region of the DEP forces. The experimental results showed that the nanogap electrode failed to block all flowing PS nanoparticles (~200 nm in diameter) when the microchannel depth was greater than 4 μm and the applied voltage was less than 5 V (Figure S5). When the microchannel depth was 2.75 μm and V*_pp_* was 5 V (10 MHz in frequency), the 200 nm PS particles suspended in fluid with the average flow velocity of 29 μm/s could be completely blocked at the immediate upstream proximity of the nanogap electrode due to the presence of a potential energy barrier (Figure S6). These results suggest that the optimal microchannel depth is a precondition for effective DEP nano-manipulation.

### 3.3. Trapping of Nanoparticles and DNAs 

**Figure 2:**
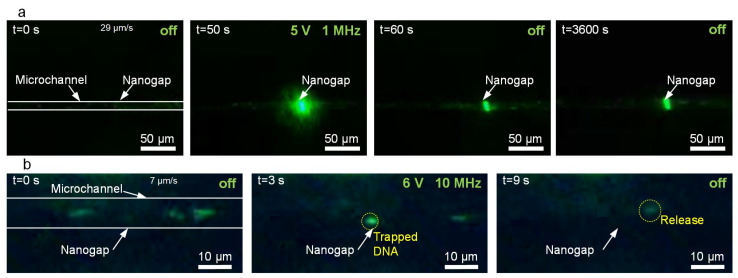
Trapping nanoparticles and biomolecules. (**a**) Trapping PS nanoparticles. (**b**) Trapping and releasing *λ-*DNA.

According to the DEP force formula presented previously, the direction and value of the DEP force depend on the CM factor, which is related to the angular frequency of the applied AC voltage and the conductivity of the suspending medium. When Re(CM) > 0, the particle experiences a P-DEP force and move to the areas with high electric field strength; conversely, when Re(CM) < 0, the particles experience an N-DEP force. Therefore, to achieve the desired trapping of nanoparticles with DEP, the frequency should be carefully adjusted. In Figure 2a, we demonstrate the capability of the nano-DEP device to trap PS nanoparticles suspended in deionized (DI) water when the frequency of the applied AC voltage (5 V) was 1 MHz. The potential energy well-generated by the P-DEP force can be calculated as ∆U_p_ = −2*πr*^3^*ε_m_*Re[CM]|**E***|*^2^ [26]. For the 200 nm PS particles, the energy well around the nanogap can be lower than −1000 *k_B_T*, where *k_B_* is the Boltzmann constant and *T* is the temperature in Kelvin, as shown in Figure S7. Such a great potential change would strongly bond the nanoparticles on the electrodes. In fact, after the trapping process for one hour, the nanoparticles were still boned on the nanogaps (Video S1), which indicates a high trapping stability of the trapping process.

To further demonstrate the trapping ability of the device, we used a P-DEP force to trap individual λ-DNA molecules (48 kb). A 0.1 μg/mL λ-DNA suspension was prepared with 1 × TE (Tris-EDTA) buffer. According to the prepared DNA concentration, the average number of DNA molecules in the observed microchannel (~100 μm long, 9 μm wide, and 2.7 μm high) was estimated to be 4.7. Because the observed number of green fluorescent dots was similar to the estimated number of DNA molecules, it is reasonable to deduce that each green fluorescent dot represents a DNA molecule. As shown in Figure 2b and Video S2, the individual strands of the YOYO-1 stained λ-DNA molecules could be captured in the solution and stably trapped at the nanogaps (input AC voltage ~ 6 V @ 10 MHz). The trapped DNA strands were immediately released when the power was switched off. When the AC voltage was applied for a long duration, the trapping of multiple DNAs could also be achieved (Video S2). These experimental results demonstrated the ability of our device to reversibly trap individual biomolecules, exhibiting its potential for single-molecule analysis.

### 3.4. Enrichment of Nanoparticles and DNAs

**Figure 3:**
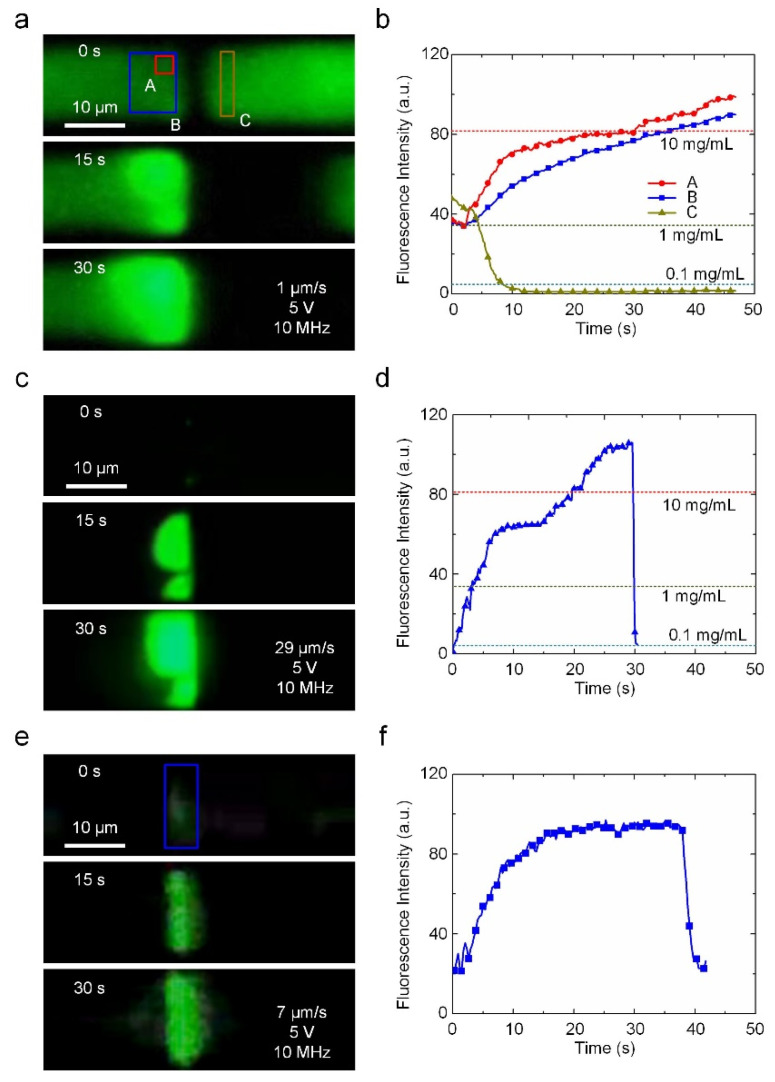
The enrichment of PS nanoparticles and DNA using N-DEP manipulation. (**a**) PS nanoparticle (initial concentration ~1 mg/mL) enrichment with N-DEP (5 V, 10 MHz). (**b**) Fluorescence intensity of the PS nanoparticles as a function of time during enrichment corresponding to (**a**). (**c**) Rapid enrichment of low-concentration PS nanoparticles (initial concentration ~0.05 mg/mL). (**d**) Fluorescence intensity vs. time of the enrichment of PS particles in (**c**). (**e**) Enrichment of λ-DNA. (**f**) Fluorescence intensity vs. time of the enrichment of DNAs.

Particle enrichment is vital for low-abundance species analysis. Due to the strong N-DEP force, the nanogap electrodes can also play an important role in enriching nanoparticles when an appropriate flow field is applied. The energy barrier generated by the N-DEP force can be calculated as ∆U_p_ = −2*πr*^3^*ε_m_*Re[CM]|**E***|*^2^ [24]. When the V*_pp_* and frequency were set to 5 V and 10 MHz, respectively, the potential energy barrier created by the N-DEP force upstream of the nanogap was approximately 46 *k_B_T* (Figure S8). Such a high potential energy barrier can prevent the PS nanoparticles (200 nm) from passing over the nanogap due to Brownian movement. Figure 3a shows that the 200 nm PS particles were successfully enriched by the N-DEP-induced damming effect. The enrichment effect was quantified by comparing the fluorescence intensity of the PS nanoparticles located at the upstream and downstream sides of the nanogap, as shown in Figure 3b. The concentration of nanoparticles at the upstream side of the nanogap increased from 1 mg/mL to 10 mg/mL in 30 s when the flow rate at the entrance was set to 1 μm/s. The concentration at the downstream side of nanogap decreased to less than 0.1 mg/mL, which confirms the strong damming effect of N-DEP manipulation. To further demonstrate the fast enrichment capabilities for low-abundance nano-species using our nano-DEP device, we also used the device to concentrate a lower concentration of PS nanoparticles with a higher flow rate (29 μm/s), as shown in Figure 3c and Video S3. The local concentration of the nanoparticles is exhibited in Figure 3d. A 200-fold enrichment was achieved in 25 s from a 0.05 mg/mL concentration. In addition, when the voltage and initial concentration were sufficiently high (10 V and 1 mg/mL), the nanoparticles could fully block up the microchannel after 30 min of enrichment (Figure S9), demonstrating the excellent enriching capability of our nano-DEP device in N-DEP mode.

The enrichment process can also be applied to biomolecules just by changing the voltage frequency. Figure 3e and Video S4 demonstrated the successful enrichment of λ-DNA (1 μg/mL). Due to the high conductivity of the medium (0.03 S/m), the frequency was set to 50 MHz to generate an N-DEP force acting on the DNA molecules. The fluorescence intensity of λ-DNA increased in the first 20 s, indicating that there was an enrichment of λ-DNAs near the nanogap due to the damming effect. However, as the concentration increased, the potential energy of the DNA aggregation also increased. Once the potential energy of the DNA aggregation exceeded the potential barrier formed by the nano-DEP, the subsequent DNA molecules passed through the nanogap without being blocked. Thus, the maximum achievable concentration of DNA molecules was limited. Notably, such a potential barrier is a function of the voltage applied to the nanogap electrodes, which causes different concentration levels. As the voltage varied from 5 V to 3 V, the fluorescence intensity decreased with the voltage, demonstrating the easy control of the local concentration by enrichment, as shown in Figure 3f.

### 3.5. Sorting of Nanoparticles and DNAs 

**Figure 4:**
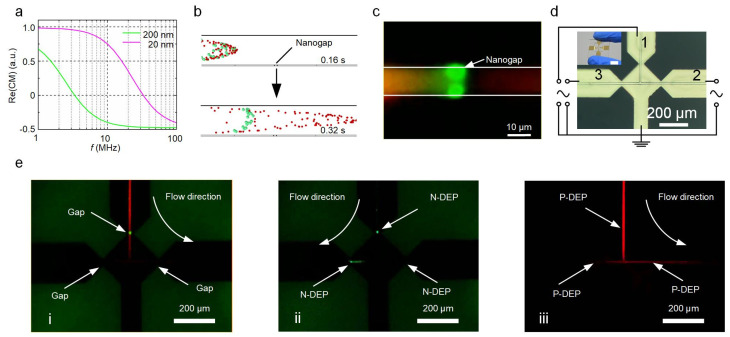
Sorting of PS nanoparticles based on size. (**a**) Plot of Re(CM) against frequency for PS nanoparticles of 200 and 20 nm in diameter. (**b**) Particle tracking simulation using COMSOL Multiphysics. (**c**) Microscopic fluorescent image of the DEP sorting process. (**d**) Three-way microchannel combined with three nanogap electrodes for the filtering of the nanoparticles. The inset shows the photograph of the three-way microchannel chip. Scale bar, 10 mm. (**e**) Microscopic fluorescent image of the filtration of nanoparticles.

Apart from the trapping and enrichment functions, the nanogap electrodes integrated with a microchannel can also be used for sorting nanoscale objects. Nanoparticles with different sizes, morphologies, or compositions generate different dielectric responses to a non-uniform electric field and are thus subject to different magnitudes and directions of DEP force, which can be employed for sorting purposes [31,38,39]. Figure 4a shows the Re(CM)–frequency curves of the 200 nm and 20 nm PS nanoparticles. There is a significant difference between the two curves, which indicates the different dielectric responses of the 200 nm and 20 nm PS nanoparticles to the same electric field and therefore enables the effective sorting of these nanoparticles through the combination of DEP force and flow field. Figure 4b is a numerical simulation showing the trajectories of both types of particles. The PS particles that were 200 nm in diameter were blocked while the 20 nm particles could pass through the nanogap electrode without any obstruction. This result was also verified by the experiment (Figure 4c and Video S5). It is noteworthy that a peak-to-peak voltage of 5 V was only able to prevent nanoparticles larger than 200 nm to cross the nanogap. For the sorting of nanoparticles smaller than 200 nm, a practical alternative is to increase the voltage and frequency to obtain a higher energy barrier and a negative value of the CM factor (Figure S10).

To further demonstrate the functionality of the nanogap electrodes, we fabricated a microfluidic device containing multiple pairs of nanogap electrodes for the filtration of nanoparticles. This device contains a three-way microchannel with each channel corresponding to a pair of nanogap electrodes (Figure 4d). Figure 4e shows the time sequence of the sorting processes for a mixture of two types of PS nanoparticles (20 and 200 nm in diameter) using the DEP manipulation method (also see Video S6). First, the mixture flowed from channel 1 to channel 2 (Figure 4e(i)). The nanoparticles with a diameter of 200 nm (in green fluorescence color) were trapped by the P-DEP force (5 V, 1 MHz) at nanogap 1, while the 20 nm particles in red fluorescence color) could freely be transported in the microchannel without hindrance (Figure 4e(ii)). Next, the AC frequency was shifted to 20 MHz and the flow direction was switched to channel 3, thus transporting a concentrated number of 200 nm particles to the left collection port and enriching them in nanogap 3. Afterward, the flow direction and frequency were switched back to run another filtration cycle (Figure 4e(iii)). During the entire filtration process, nanogap 2 operated in damming mode to verify whether there were any 200 nm nanoparticles transported to channel 2. By repeating the above process, the 200 nm particles were removed as impurities while the 20 nm particles were filtered from the suspension.

**Figure 5:**
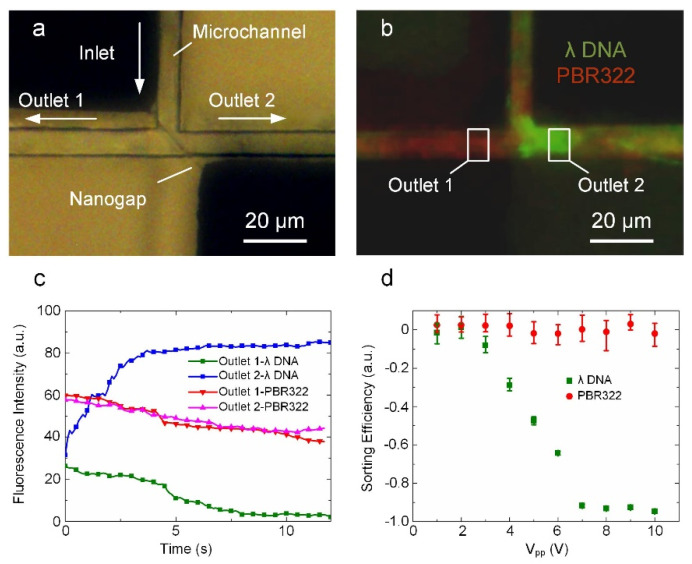
Size-based sorting of DNA molecules using the nano-DEP device. (**a**) A bright microscopic image showing a T-shape microchannel bonded on the nanogap electrodes. (**b**) A microscopic fluorescent image showing the sorting of YOYO-1 stained λ-DNA (green) and POPO-3 stained PBR322 (red) with the nano-DEP device. (**c**) Fluorescence intensities versus time in the sorting process. (**d**) Sorting efficiency of λ-DNA and PBR322 at different AC voltages.

In addition to the sorting of nanoparticles, we also applied the nanogap-electrode-based DEP device to continuously sort different sizes of DNA molecules. The key structure of the nano-DEP device used for size-based sorting of DNA molecules is shown in Figure 5a and comprises a T-shaped microchannel integrated with a slanted nanogap electrode at the junction. The slanted nanogap provides an outlet for the larger DNAs without changing the voltage on the nanogaps. In this DNA sorting experiment, the sample was a mixture of YOYO-1 stained λ-DNA (48.5 kbp, 10 μg/mL) and POPO-3 stained PBR322 (4.26 kbp, 10 μg/mL) at a ratio of 1:2 and the flow rate was set at approximately 7 μm/s. As previously mentioned, when a proper AC voltage is applied to the nanogap electrodes during the sorting process, an energy barrier is generated to prevent DNA molecules from passing through the nanogap. Since the energy barrier acting on a particle is proportional to the cube of the size of the particle, larger particles experience a much higher energy barrier compared with smaller particles. As shown in Figure 5b, in the case of a tailored AC voltage (8 V, 50 MHz) applied to the nanogap electrodes, the λ-DNA molecules (green) experience a potential energy barrier substantially higher than their kinetic energy and is prevented from flowing over the nanogap. In contrast, the PBR322 DNA molecules (red) experience a substantially lower potential energy barrier due to their smaller size and can pass over the nanogap. Thus, under the action of a hydrodynamic drag force, the blocked λ-DNA molecules are selectively diverted into outlet 2 and the PBR322 DNA molecules are equally diverted into outlets 1 and 2 (Video S7). 

To evaluate the sorting efficiency, we measured the fluorescence intensities of the sample at the two outlets. As shown in Figure 5c, there is an increase in the λ-DNA fluorescence intensity at outlet 2, but a decrease for that at outlet 1, indicating that a strong damming effect is exerted on the λ-DNA molecules. In contrast, the PBR322 fluorescence intensity at both outlets showed the same trend, indicating that almost no potential energy barrier was present for the PBR322 DNA molecules. It is noted that the decrease in the red fluorescence intensities can be attributed to the fluorescence attenuation under strong illumination. The bimodal sorting efficiency is defined as [39]:

(1) $$\sum *=\frac{I_{1}-I_{2}}{I_{1}+I_{2}}$$

where *I*_1,2_ represent the fluorescence intensities at outlet 1 and outlet 2, respectively. The sorting efficiency can range from −1 to 1. A sorting efficiency of close to 1 indicates that the particles were likely to flow to outlet 1. Additionally, a sorting efficiency close to −1 implies a higher concentration at outlet 2. According to DEP theory, the DEP force is proportional to the square of the electric field intensity. As expected, higher voltages lead to higher sorting efficiencies (as shown in Figure 5d). When the applied V*_pp_* was larger than 7 V, the sorting efficiency of λ-DNA exceeded −0.92. In contrast, when the applied V*_pp_* was less than 3 V, the sorting efficiency of λ-DNA was close to 0. These results demonstrate that our nano-DEP device can achieve efficient size-based sorting of DNA in a continuous manner by tailoring the applied AC voltage. Such nano-DEP devices offer several unique advantages for DNA sorting applications including short processing times, small sample volumes, and good portability.

## 4. Conclusions

We demonstrated a nanogap-electrode-enabled electrokinetic manipulation scheme of nanoscale objects in a fluidic environment. Powered by a strong electric field and the associated field gradient in the vicinity of the nanogap electrodes, this DEP platform can efficiently manipulate nanoscale objects with a low voltage without the issue of the Joule heating effect. In addition, the tunability of the amplitude and frequency of the applied voltages enables this DEP electrokinetic manipulation to be operated in two different modes: damming and trapping, providing great flexibility, and increased functionality for the manipulation of one or multiple nanoscale objects. By using the damming and trapping effects, we successfully demonstrated the capturing, enriching, and sorting of nanoparticles and DNA molecules in a microfluidic environment. More importantly, our nano-DEP devices can be fabricated with conventional lithography, which makes their production economical, facilitating the accessibility of nano-manipulation to more users in various fields. The versatility of this nano-DEP device, along with its simplicity of fabrication, renders it a promising tool for biomedical applications including (1) the capture and immobilization of a single biomolecule for further electrical and optical characterization; (2) the concentration of low abundance biospecies for highly sensitive detection, which is useful in early disease diagnostics; and (3) the sorting and filtering of target biospecies from complex samples for further analysis. We believe that the electrokinetic manipulation demonstrated in this paper will help to bridge the gap between nano-manipulation and microfluidics.

## Data Availability

Not applicable.

## Supplementary Materials

The following supporting information can be downloaded at: https://www.mdpi.com/article/10.3390/bios12070451/s1, Note S1: DEP induced nanoparticle speed; Note S2: ACEO and ACET flow [40,41,42,43]; Figure S1: The structure of the whole readout system; Figure S2: Illustration of the PDMS microchannel preparation process; Figure S3: The electric field around nanogap; Figure S4: The flow rate in the microchannel; Figure S5: Theoretical analysis and experimental investigation of the effect of the applied voltage value on DEP manipulation in a 4.1 μm deep channel; Figure S6: Theoretical analysis and experimental investigation of the effect of the applied voltage value on DEP manipulation in a 2.75 μm deep channel; Figure S7: The potential well generated by P-DEP manipulation; Figure S8: The potential barrier generated by N-DEP manipulation; Figure S9: Blocked microchannel after the N-DEP (10 V, 10 MHz) process and continuously flowing nanoparticle solution from left to right (29 μm/s) for 30 min; Figure S10: The simulation of nanoparticle movement around nanogaps with different particle sizes; Video S1: The trapping of PS nanoparticles was conducted by an AC voltage (5 V, 1 MHz); Video S2: The trapping of λ-DNA was conducted by an AC voltage (6 V, 10 MHz); Video S3: PS nanoparticle solution (200 nm, 0.05 mg/mL) was used for the enrichment performance testing of nanogap electrodes; Video S4: The enrichment of λ-DNA (mg/mL) was conducted by an AC voltage (5 V, 50 MHz); Video S5: Controlled sorting of PS nanoparticles with the diameter of 200 nm and 20 nm by N-DEP (5 V, 40 MHz, flow rate, 1 μm/s); Video S6: The sorting of PS nanoparticles by a 3-way channel; Video S7: The separation of λ-DNA and PBR322 is shown in this video (8 V, 50 MHz).
