# Supplementary material for: Nanogap Electrode-Enabled Versatile Electrokinetic Manipulation of Nanometric Species in Fluids
Source: Biosensors (Basel). 2022 Jun 24;12(7):451. doi: 10.3390/bios12070451 (PMC9313323; doi:10.3390/bios12070451)
Supplement: Supplementary file 1 [file biosensors-12-00451-s001.zip › biosensors-1775522-supplementary.pdf]

Nanogap Electrode-Enabled Versatile Electrokinetic Manipulation of Nanometric Species in Fluids

Qiang Zhao‡[1], Yunjiao Wang‡[2], Bangyong Sun[1], Deqiang Wang[2,*] and Gang Li[1,*].

[1] Key Laboratory of Optoelectronic Technology and Systems, Ministry of Education, Defense Key Disciplines Lab of Novel Micro-Nano Devices and System Technology, Chongqing University, Chongqing, 400044, China.

[2] Chongqing Key Laboratory of Multi-Scale Manufacturing Technology, Chongqing Institute of Green and Intelligent Technology, Chongqing 400714, China.

Note S1. DEP Induced Nanoparticle Speed

For a spherical particle, the time-averaged dielectrophoretic force acting on the nanoparticles can be expressed as

$$\mathbf{F}_{DEP} = 2\pi\varepsilon_f r^3 \,\mathrm{Re}(\mathrm{CM})\nabla\mathbf{E}^2 \tag{1}$$

where ε_f is the dielectric constant of water ($78\varepsilon_0$, $\varepsilon_0 = 8.85 \times 10^{-12}$ F/m). The Clausius–Mosotti factor depends on the frequency and can be given as

$$\mathrm{CM} = \frac{\tilde{\varepsilon}_p - \tilde{\varepsilon}_f}{\tilde{\varepsilon}_p + 2\tilde{\varepsilon}_f} \tag{2}$$

where $\tilde{\varepsilon}_i = \varepsilon_i - j(\sigma_i / \omega)$ is the complex permittivity of a lossy dielectric. Subscript p represents the particle. ε_i and σ_i are the permittivity and conductivity of the particles and fluids, respectively. The Re(CM) is hence given by

$$\mathrm{Re(CM)} = \frac{(\varepsilon_p - \varepsilon_f)(\varepsilon_p + 2\varepsilon_f) + (\sigma_p - \sigma_f)(\sigma_p + 2\sigma_f)/\omega^2}{(\varepsilon_p + 2\varepsilon_f)^2 + (\sigma_p + 2\sigma_f)^2/\omega^2} \tag{3}$$

In this work, the σ_f was measured by a conductivity analyzer ($\sigma_f \approx 0.001$ S/m, DDS-11(A) Inesa Scientific Instrument Co. Ltd). The permittivity of the PS particles was 2.55. Due to the surface conductance, the equivalent conductivity was associated with the radius of PS particles ($\sigma_p = \sigma_b + 2K_s/\mathrm{r}$, $\sigma_b \approx 0$, $K_s = 1$ nS)[1]. Based on **Equations (1)–(3)**, the $\mathbf{F}_{\mathrm{DEP}}$ on nanoparticles can be obtained by numerical simulation. The relative motion between spherical nanoparticles and fluid can be given by[2]

$$\mathbf{u}_{\mathrm{DEP}} = \frac{\mathbf{F}_{\mathrm{DEP}}}{6\pi r\eta} \tag{4}$$

therefore, the total particle speed can be given by

$$\mathbf{u}_{\mathrm{Total}} = \mathbf{u}_{\mathrm{DEP}} + \mathbf{u}_{\mathrm{Fluid}} \tag{5}$$

Note S2. ACEO and ACET Flow

When an AC voltage is applied to electrodes, the time-averaged slip velocity at the surface of the electrodes driven by an electric field is[3]

$$\langle \mathbf{u}_{slip} \rangle = -\frac{\varepsilon_f \langle \zeta \mathbf{E}_t \rangle}{\eta} = \frac{1}{2}\frac{\varepsilon_f}{\eta}\frac{1}{1+\delta}\mathrm{Re}\left[(\tilde{\phi} - \tilde{V}_0)(\tilde{\mathbf{E}} - \tilde{\mathbf{E}}\cdot\mathbf{n}\cdot\mathbf{n})^*\right] \tag{6}$$

where $\mathbf{E}_{\mathrm{t}} = \mathbf{E} - \mathbf{E}\cdot\mathbf{n}\cdot\mathbf{n}$ is the tangential of the electric field component of the electric field; η is the dynamic viscosity of water (0.001Pa·s); $\zeta = \frac{1}{1+\delta}(V_0 - \phi)$ is the zeta

potential of the electrical double layer; $\delta = \frac{C_d}{C_s}$ is the capacitance ratio of the diffuse layer to the stern layer; $C_d = \frac{\varepsilon_f}{\lambda_d}$ is the capacitance of diffuse layer; $\lambda_d = \sqrt{\frac{\varepsilon_f D}{\sigma_f}}$ is the double-layer thickness; D is the diffusion coefficient of the ions (assumed to be 2×10^{-9} m^2/s); C_s is the capacitance of stern layer (assumed to be 0.8 F/m^2); V_0 is the transient potential value of the electrodes; ϕ is the bulk potential outside the electrical double layer; tilde mark is the complex function of sine signal ($\tilde{\phi} = A_0 e^{i\varphi}$, A_0 is the amplitude, and φ is the phase angle); the asterisk is a complex conjugate operator. In this simulation, the bulk potential ϕ is obtained by the frequency domain model built into COMSOL Multiphysics. A contact impedance layer was set on the surface of the electrodes with a surface capacitance of $C_0 = \frac{C_d C_s}{C_d + C_s}$.

The ACET flow in the microchannel induced the interaction between the electric field and variation in the conductivity and permittivity due to the Joule heating. The temperature distribution around the nanogap can be calculated by the following equations [4, 5]

$$-k\nabla^2 T + \rho C_p \mathbf{u}\nabla T = \frac{\sigma_f}{2}|\mathbf{E}|^2 \tag{7}$$

where $\frac{\sigma_f}{2}|\mathbf{E}|^2$ represents the Joule heating generated by electric fields; σ_f is the conductivity of water; k, ρ, and C_p is the thermal conductivity, density, and thermal capacity of water, respectively; T is the temperature; $\mathbf{u}$ is the velocity of the fluid in microchannel.

Due to the temperature variation in the microchannel, the local charge density ρ_e can be estimated by[6]

$$\rho_e = \varepsilon_f \left(\frac{1}{\varepsilon_f}\frac{\partial \varepsilon_f}{\partial T} - \frac{1}{\sigma_f}\frac{\partial \sigma_f}{\partial T} \right) \frac{\nabla T.\mathbf{E}}{1 + i\omega\tau} \tag{8}$$

where ω is the applied voltage angular frequency; j is the imaginary number; $\tau = \varepsilon_f/\sigma_f$ is the charge relaxation time. In aqueous solutions, the temperature dependence of the permittivity and conductivity can be estimated as $\alpha = \frac{1}{\varepsilon_f}\frac{\partial \varepsilon_f}{\partial T} = -0.004\mathrm{K}^{-1}$ and $\beta = \frac{1}{\sigma_f}\frac{\partial \sigma_f}{\partial T} = 0.02\mathrm{K}^{-1}$. The volumetric force F_{ET} on the fluid is given by

$$\mathbf{F}_{ET} = \rho_e \mathbf{E} - \frac{1}{2}|\mathbf{E}^2|\nabla \varepsilon_f \tag{9}$$

With **Equations (7)–(9)**, the F_{ET} is hence given by

$$\mathbf{F}_{ET} = 0.5\varepsilon_f \left(-0.024 \bullet \mathrm{K}^{-1} \times \frac{\nabla T.\mathbf{E}}{1+(\omega\tau)^2}\mathbf{E} + 0.002 \bullet \mathrm{K}^{-1} \times \left|\mathbf{E}^2\right| \nabla T \right) \tag{10}$$

The two terms in **Equation (10)** represent the Coulomb part and the dielectric part of $\mathbf{F}_{ET}$, respectively. In low frequency, $\frac{1}{1+(\omega\tau)^2} \approx 1$, the coulomb part is the dominant factor. When the frequency is much higher than τ^{-1}, $\mathbf{F}_{ET}$ appears as a reversed variation.

In the COMSOL Multiphysics simulation, the entrance and exit were set as the ambient temperature (293.15 K). The thermal conductivity of water, PDMS, and epoxy were set as 0.6, 0.2, and 0.9 W/(m·K), respectively. The thermal conductivities of Cr and Au were obtained from COMSOL Multiphysics. The nonslip boundary condition was applied to the surface of the microchannel.

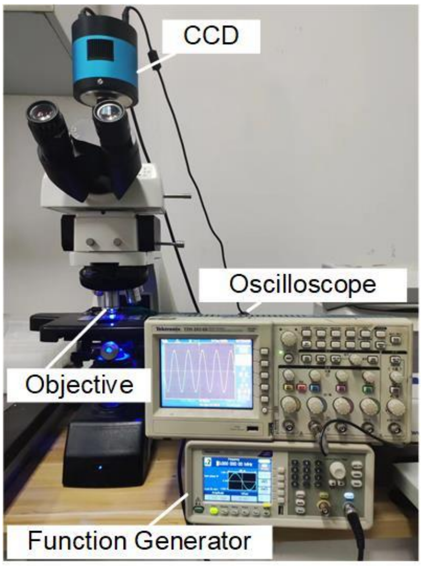

Figure S1. The setup of the system for performing and recording the nanogap electrode-enabled dielectrophoretic manipulation of nanoparticles.

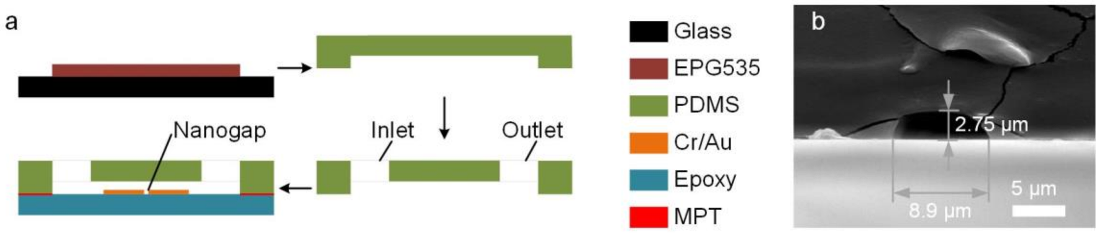

Figure S2. Illustration of the PDMS microchannel preparation process. (**a**) An EPG 535-based mold was photolithographically patterned, and then PDMS was cast on the mold to produce microchannels. Reservoirs were made at the end of each channel by punching, and the punched PDMS slab was then aligned and bonded to the epoxy substrate containing nanogap electrodes. (**b**) The SEM cross-sectional image of a microchannel. The microchannel was measured as 8.9 μm in width and 2.75 μm in depth. Due to the repeated mold transfer process, the microchannel had rounded corners. The thin electrode layer (100 nm) was invisible in this image.

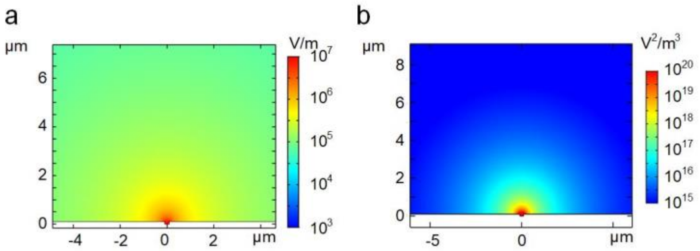

Figure S3. The electric field around nanogap. (**a**) The electric field strength around the nanogap. (**b**) The $\nabla \mathbf{E}^2$ around the nanogap. The gap width and V_{pp} were set as 200 nm and 5 V, respectively.

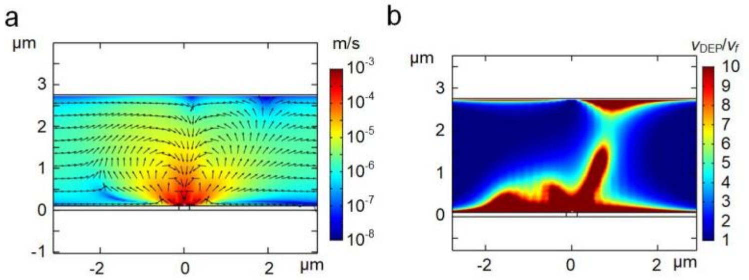

Figure S4. The flow rate in a microchannel. (**a**) The side view of the ACEO and ACET flow (2 V, 100 kHz). The color map and arrows represent the rate and direction of the flow. Two vortexes were observed around the nanogap. (**b**) The comparison between the flow rate of the fluid and the DEP manipulation of 200 nm PS particles. The flow rate of the fluid was lower than the DEP-induced particle speed around the nanogaps.

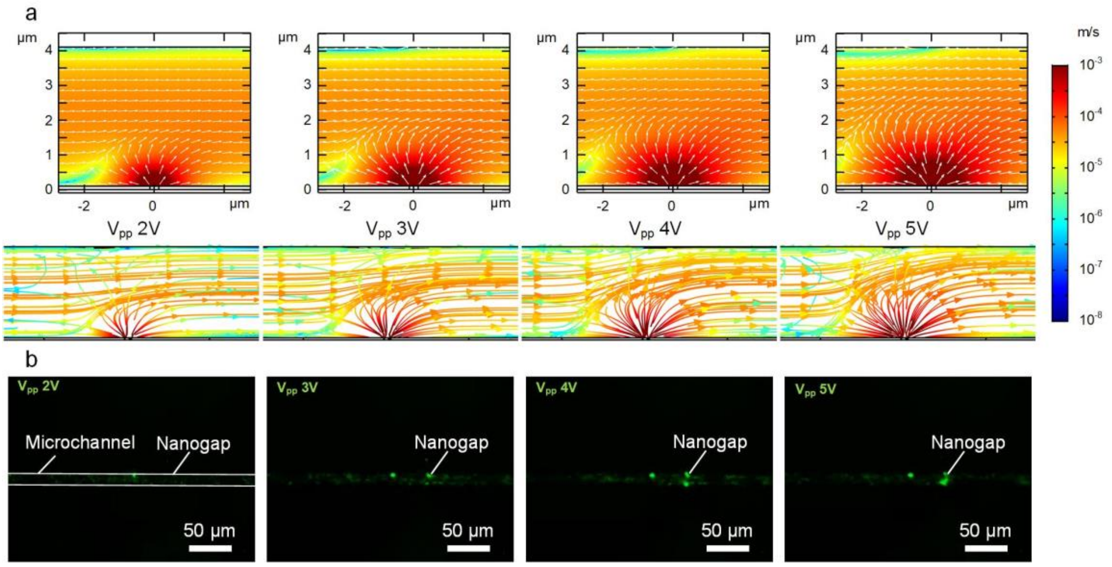

Figure S5. Theoretical analysis and experimental investigation of the effect of the applied voltage value on DEP manipulation in a 4.1 μm deep channel. (**a**) The velocity vectors and streamlines of PS nanoparticles (0.01 mg/mL dispersed in water) in a microchannel. The frequency, fluid velocity, and the AC voltage applied on the nanogap were 10 MHz, 29 μm/s, and 2–5 V., respectively. (**b**) The experimental results of the nanoparticles manipulated by N-DEP in a 4.1 μm deep channel. Both the simulation and experiment showed limited obstruction at the bottom of the microchannel.

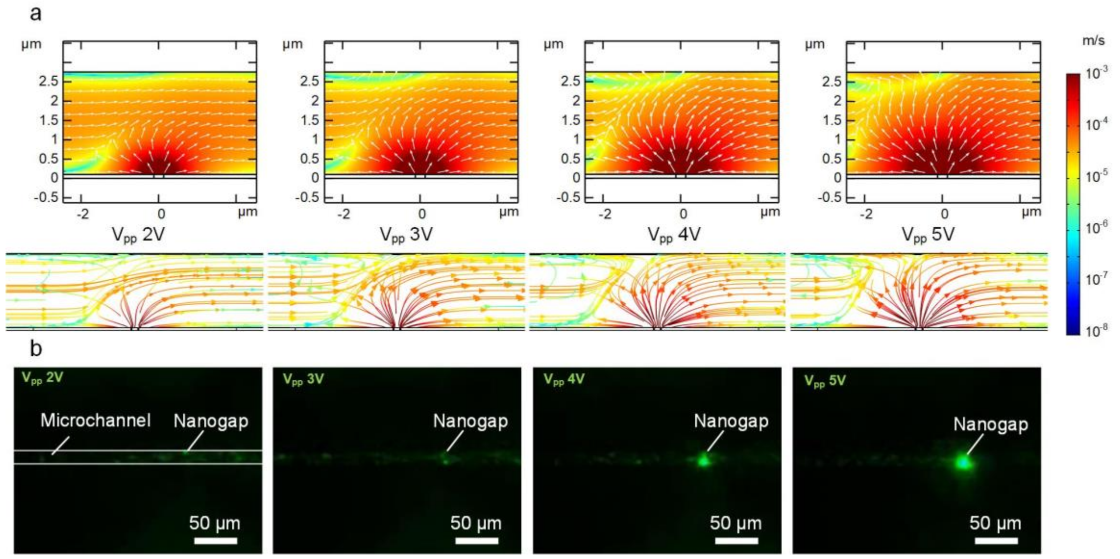

Figure S6. Theoretical analysis and experimental investigation of the effect of the applied voltage value on DEP manipulation in a 2.75 µm deep channel. (**a**) Diagrams of the velocity vectors and streamlines of the PS nanoparticles (0.01 mg/mL dispersed in water) of 200 nm in diameter. The AC voltage applied on the nanogap is in the range of 2 V to 5 V. The frequency and fluid velocities were 10 MHz and 29 µm/s. Voltage lower than 4 V was unable to prevent the nanoparticles from getting through. (**b**) The experimental results of the green fluorescence-labeled nanoparticles manipulated by N-DEP.

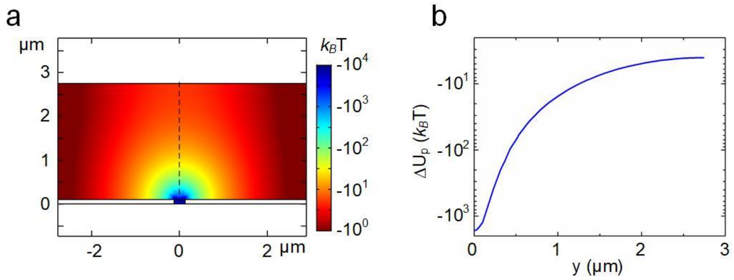

Figure S7. The potential well generated by DEP manipulation. (**a**) The potential change around the nanogap in the nanoparticle trapping process (5 V, 1 MHz). (**b**) The potential change along the dashed line in Figure S3a.

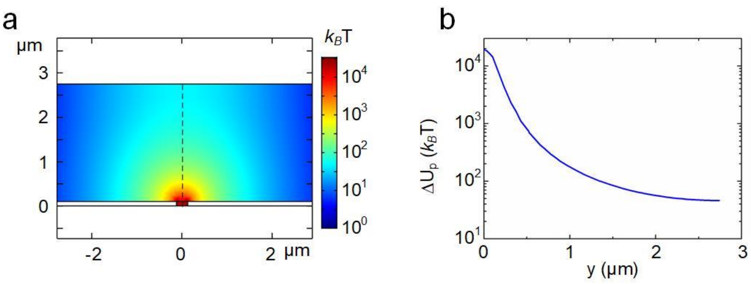

Figure S8. The potential barrier generated by DEP manipulation. (**a**) The energy barrier of N-DEP around the nanogap (5 V, 10 MHz). (**b**) Potential energy versus position y along the symmetry of the nanogap. The lowest barrier was located at the top of the microchannel (46 k_BT).

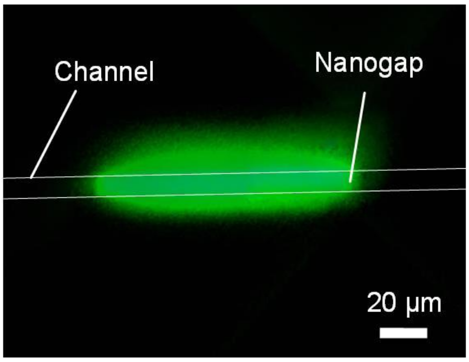

Figure S9. Blocked microchannel after the N-DEP (10 V, 10 MHz) process and continuously flowing nanoparticle solution from left to right (29 μm/s) for 30 min. The initial concentration of the nanoparticles was 1 mg/mL. Nanoparticles were confined to the left of the nanogap and blocked the microchannel. The subsequent nanoparticles were squeezed by the flow and resulted in a long blocked microchannel (106 μm).

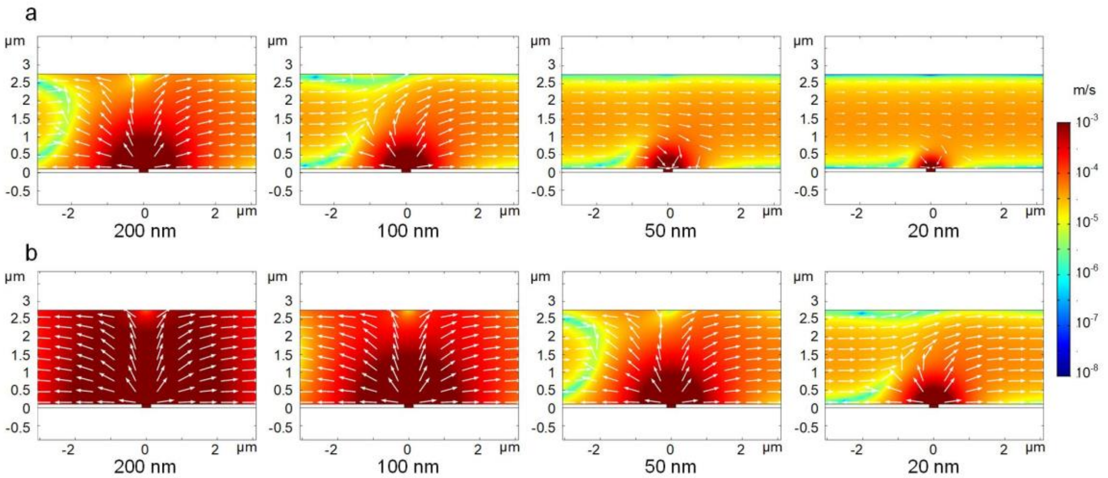

Figure S10. The simulation of the nanoparticle movement around the nanogaps with different particle sizes. (**a**) The movement of nanoparticles (200, 100, 50, 20 nm) when the AC voltage was applied on the nanogap was 20 MHz, 5 V. (**b**) The movement of the nanoparticles (200 nm) when the AC voltage was applied on the nanogap was 20 MHz, 20 V.

References

1 I. Ermolina; H. Morgan. The electrokinetic properties of latex particles: comparison of electrophoresis and dielectrophoresis, *J. Colloid Interf. Sci.,* **2005,** 285, 419-428.

2 E. Yu, *et al*. Precise capture and dynamic relocation of nanoparticulate biomolecules through dielectrophoretic enhancement by vertical nanogap architectures, *Nat. Commun.,* **2020,** 11, 2804.

3 Y. Ren, *et al*. Induced-charge electroosmotic trapping of particles, *Lab Chip,* **2015,** 15, 2181-2191.

4 B. H. Lapizco-Encinas. On the recent developments of insulator-based dielectrophoresis: A review, *Electrophoresis,* **2019,** 40, 358-375.

5 M. R. Hossan; D. Dutta; N. Islam; P. Dutta. Review: Electric field driven pumping in microfluidic device, *Electrophoresis,* **2018,** 39, 702-731.

6 W. Liu, *et al*. A theoretical and numerical investigation of travelling wave induction microfluidic pumping in a temperature gradient, *Journal of physics. D, Applied physics,* **2014,** 47, 75501.
